# Nutrient stoichiometry and land use rather than species richness determine plant functional diversity

**DOI:** 10.1002/ece3.3609

**Published:** 2017-12-03

**Authors:** Verena Busch, Valentin H. Klaus, Caterina Penone, Deborah Schäfer, Steffen Boch, Daniel Prati, Jörg Müller, Stephanie A. Socher, Ülo Niinemets, Josep Peñuelas, Norbert Hölzel, Markus Fischer, Till Kleinebecker

**Affiliations:** ^1^ Institute for Landscape Ecology Westfälische Wilhelms‐Universität Münster Muenster Germany; ^2^ Institute for Agricultural Sciences, Grassland Sciences ETZ Zürich Zürich Switzerland; ^3^ Institute of Plant Sciences University of Bern Bern Switzerland; ^4^ Swiss Federal Research Institute WSL Birmensdorf Switzerland; ^5^ Institute of Biochemistry and Biology University of Potsdam Potsdam Germany; ^6^ Institute for Ecology and Evolution University of Salzburg Salzburg Austria; ^7^ Department of Plant Physiology Estonian University of Life Sciences Tartu Estonia; ^8^ Global Ecology Unit CREAF‐CSIC Universidad Autónoma de Barcelona Barcelona Spain; ^9^ CREAF Cerdanyola del Vallès Spain

**Keywords:** biodiversity exploratories, fertilization, leaf economics, mowing, nutrient availability, nutrient ratios, phosphorus, plant functional traits, plant strategies, seed mass

## Abstract

Plant functional traits reflect individual and community ecological strategies. They allow the detection of directional changes in community dynamics and ecosystemic processes, being an additional tool to assess biodiversity than species richness. Analysis of functional patterns in plant communities provides mechanistic insight into biodiversity alterations due to anthropogenic activity. Although studies have consi‐dered of either anthropogenic management or nutrient availability on functional traits in temperate grasslands, studies combining effects of both drivers are scarce. Here, we assessed the impacts of management intensity (fertilization, mowing, grazing), nutrient stoichiometry (C, N, P, K), and vegetation composition on community‐weighted means (CWMs) and functional diversity (Rao's *Q*) from seven plant traits in 150 grasslands in three regions in Germany, using data of 6 years. Land use and nutrient stoichiometry accounted for larger proportions of model variance of CWM and Rao's *Q* than species richness and productivity. Grazing affected all analyzed trait groups; fertilization and mowing only impacted generative traits. Grazing was clearly associated with nutrient retention strategies, that is, investing in durable structures and production of fewer, less variable seed. Phenological variability was increased. Fertilization and mowing decreased seed number/mass variability, indicating competition‐related effects. Impacts of nutrient stoichiometry on trait syndromes varied. Nutrient limitation (large N:P, C:N ratios) promoted species with conservative strategies, that is, investment in durable plant structures rather than fast growth, fewer seed, and delayed flowering onset. In contrast to seed mass, leaf‐economics variability was reduced under P shortage. Species diversity was positively associated with the variability of generative traits. *Synthesis*. Here, land use, nutrient availability, species richness, and plant functional strategies have been shown to interact complexly, driving community composition, and vegetation responses to management intensity. We suggest that deeper understanding of underlying mechanisms shaping community assembly and biodiversity will require analyzing all these parameters.

## 1 INTRODUCTION

Mankind has had great impact on the global environment since Neolithic times, altering climate, topography, mobility of biota, and biogeochemical cycles (Chapin et al., 2000).

Biodiversity has consequently decreased at exceptionally high rates (Pimm, Russel Gareth, Gittleman John, & Brooks, 1995), which is expected to continue with land‐use change as a main driver (Chapin et al., 2000; Sala, 2000).

In central European seminatural grasslands, land‐use change in form of homogenization, cessation, and/or intensification of traditional land use, has caused notable decreases in biodiversity especially since the 1950s (de Bello et al., 2010; Hodgson et al., 2005; Poschlod, Bakker, & Kahmen, 2005). Specifically, the increase in agricultural management intensity has influenced vegetation patterns, composition, and dynamics through increased vegetation disturbance (mowing, grazing) and altered soil fertility (fertilization) (Louault, Pillar, Aufrère, Garnier, & Soussana, 2005). Generally, plant species assemble and coexist along land‐use gradients occupying all available ecological niches based on their responses to disturbance and their traits and strategies for acquiring resources. In intensively managed ecosystems, where disturbance is frequent and nutrient availability is high, disturbance‐tolerant species that readily absorb nutrients and quickly regrow after biomass removal, outgrow and outcompete less tolerant or less competitive species. In less intensively managed or unmanaged ecosystems, disturbance frequency and nutrient availability are low, allowing only for species to persist that are able to cope with nutrient stress and lack of biomass removal. Moderately managed ecosystems, characterized by moderate disturbance frequencies and intermediate nutrient availability levels, are able to maintain highest levels of biodiversity (“Intermediate disturbance hypothesis”); a pattern that, along a land‐use gradient, results in a “hump‐shaped” curve of species richness (Grime, 1979).

Biodiversity, however, is not only represented by species richness, but includes aspects ranging from genetic diversity within populations to species and community diversity and processes in ecosystems and across landscapes (Chapin et al., 2000; Sala, 2000). Functional composition and diversity of species communities are important dimensions of biodiversity and are increasingly used to identify processes of ecosystems and species assembly (Fortunel et al., 2009; Lavorel & Garnier, 2002; Westoby, Falster, Moles, Vesk, & Wright, 2002). Functional composition and diversity can provide clearer insight into the mechanisms driving local changes in vegetation, biodiversity dynamics, and ecosystemic processes than traditional diversity indices of species richness (Díaz et al., 2007; Hillebrand & Matthiessen, 2009; Hooper et al., 2005; Pfestorf et al., 2013).

Land use affects functional composition on one hand and drives plant functional diversity in grasslands on the other hand. Fertilization and disturbance intensity, for instance, have been closely linked to these functional indices before (Hooper et al., 2005; Pakeman, 2011; Sala, 2000; Socher et al., 2013), and their effects have been well studied (e.g., Díaz et al., 2007; Garnier et al., 2007; Laliberte et al., 2010). Fertilization, for example, favors species with high levels of vegetative growth and seed output (Lavorel & Garnier, 2002), whereas disturbance, such as mowing or herbivory, tend to select for short, rosette‐forming species producing either many small or few large diaspores (Díaz et al., 2007; Lienin & Kleyer, 2011). In contrast, decreasing land use can lead to the dominance of both tall‐ and small‐growing plants with conservative traits of foliar economy such as low‐specific leaf area (SLA), high leaf dry matter content (LDMC), and delayed flowering (Garnier et al., 2007; McIntyre, 2008).

Grassland productivity is limited by the availabilities of nitrogen (N), but also phosphorus (P) and occasionally potassium (K) (Güsewell, 2004; Olde Venterink, Wassen, Verkroost, & de Ruiter, 2003; Sardans & Peñuelas, 2015), elements which, among other factors, drive grassland community assembly and structure (Daufresne & Hedin, 2005; Fay et al., 2015; Koerselman & Meuleman, 1996). The intensification of land use has possibly shifted patterns of nutrient availability and use, as it has led to large‐scale N eutrophication and thus potentially fueling P and K limitation (Mahowald et al., 2008). The ratios of these elements in foliar biomass can be used as indicators of relative availability in the soil (nutrient stoichiometry), pinpointing the nature of nutrient limitation and thus the regulation of productivity at community level (Klaus et al., 2011; Marschner, 2012).

The changes in soil fertility and productivity due to land‐use practices and the relationships between nutrient stoichiometry and community assembly, composition and change in terrestrial systems, have been well studied (Güsewell, 2004; Güsewell & Koerselman, 2002; Niinemets & Kull, 2005; Olde Venterink et al., 2003). For example, nutrient‐poor, low‐disturbance, and species‐rich grasslands can support perennial plants with nutrient‐retentive strategies (Pakeman, Lepš, Kleyer, Lavorel, & Garnier, 2009), whereas resource‐rich environmental conditions favor species‐poorer communities composed of competitive species specializing in nutrient acquisition and productivity (Lienin & Kleyer, 2011). The interplay of competitive and stress tolerance strategies can be reflected in foliar‐economic, generative, and phenological traits (Velbert, Kleinebecker, Mudrak, Schwartze, & Hölzel, 2017).

Nutrient ratios inferred from foliar concentrations as indicators for nutrient availability/limitation, however, have rarely been included in studies of functional traits (Güsewell, 2004; Nikolic, Bocker, Kostic‐Kravljanac, & Nikolic, 2014), although the combination of both aspects may be particularly useful for identifying the consequences of land‐use change (Fujita et al., 2014).

Here, we assessed the effects of land use (intensities of fertilization, mowing, and grazing), nutrient stoichiometry, major soil characteristics, species richness, and community composition on functional composition and diversity across a wide range of grasslands. We also assessed and compared the relative importance (explained variance) of single factors on functional composition and diversity for identifying overall patterns and the main driving mechanisms.

We hypothesized that

1. Land use is the most important driver of both functional composition and diversity of all trait groups analyzed.We expect a positive correlation between land‐use intensity and leaf‐economy and generative traits, because species favored by intensive land use are expected to compensate the effects of mowing and grazing by higher investments in vegetative growth and seed output.
2. Nutrient stoichiometry plays an important role in leaf economic and generative traits, but a minor one in functional diversity.We expect a high investment in traits associated with competition under high nutrient availability and stronger investment in traits associated with stress tolerance under nutrient limitation. We anticipate increases in functional diversity under nutrient limitation, as species need to exploit the little amounts of resources available by niche partitioning; and decreased functional diversity under high nutrient availability as few competitive species need to compartmentalize available resources.
3. Plant species diversity is mainly reflected by diversity in leaf economics and generative traits. We expect larger investment in fast growth, biomass production and high‐seed output in intensively used, species‐poor, and functionally similar communities. Plants in less intensively used, species‐rich and functionally diverse communities should invest in the production of durable tissue, slow growth, and large seeds.

## 2 METHODS

### 2.1 Study area

Our study was part of the framework of the Biodiversity Exploratories, a large‐scale and long‐term research project studying functional biodiversity (Fischer et al., 2010). We studied grasslands in three regions in Germany (Table 1); the UNESCO Schorfheide‐Chorin Biosphere Reserve, the Hainich National Park and its surroundings, and the Schwäbische Alb Biosphere Area. The relatively evenly distributed sites were variously managed and represented a gradient of land‐use intensity typical for most central European agricultural grasslands (Fischer et al., 2010).

**Table 1:** Main geographic and environmental characteristics of the three Biodiversity Exploratories. Taken from Fischer et al. (2010)

	Schorfheide‐Chorin	Hainich‐Dün	Schwäbische Alb
Location	NE Germany	Central Germany	SW Germany
Size	ca. 1,300 km	ca. 1,300 km	ca. 422 km^2^
Geology	Young glacial landscape	Calcareous bedrock	Calcareous bedrock, karst phenomena
Altitude a.s.l.	3–140 m	285–550 m	460–860 m
Annual mean temperature	8–8.5°C	6.5–8°C	6–7°C
Annual mean precipitation	500–600 mm	500–800 mm	700–1,000 mm

### 2.2 Study design and land‐use intensity

We studied 50 relatively evenly distributed grassland plots in each of the regions, comprising agricultural meadows, pastures (grazed by sheep, goats, horses, or cattle) or mown pastures along a gradient of land‐use intensity. Soil types were categorized as mineral or organic, and soil depths and pH values were obtained from the soil inventory data set (Fischer et al., 2010). Land‐use information for each plot was obtained annually from a standardized questionnaire filled in by farmers and land owners. An index of continuous land‐use intensity (LUI) proposed by Blüthgen et al. (2012) was calculated for each year for quantitatively assessing the variation of each of the land‐use components (fertilization [N kg × ha^−1^ × year^−1^], mowing [1 to 4 cuts per year] and grazing [LU × days × ha^−1^ × year^−1^]) and for reducing the complexity of management complexity to one dimension. For this, the globally standardized sum of each land‐use component (fertilization, mowing, grazing), relative to its mean within the corresponding region, was root transformed for a more even distribution (Blüthgen et al., 2012). For each experimental plot I, the land‐use intensity *L*_*i*_ is defined as

$$L_{i}=F_{i}\times F_{R}^{-1}+M_{i}\times M_{R}^{-1}+G_{i}\times G_{R}^{-1},$$

where *F*_*i*_ is the fertilization level, *M*_*i*_ the mowing frequency per year and *G*_*i*_ the grazing intensity on each site *i* for a given year; and *F*_R_, *M*_R_, and *G*_R_ their respective mean within its region R for that year.

### 2.3 Sampling and stoichiometric analyses

The vegetation in a 4 m × 4 m quadrat was recorded annually from mid‐May to mid‐June from 2008 to 2013. Species number and coverage, coverage of litter, open soil, moss and lichens, stones, and woody parts were estimated in percent. Aboveground community biomass was sampled at the same time by cutting the vegetation at a height of 2–3 cm in four 0.5 × 0.5 m subplots. The biomass was dried at 80°C for 24 hr, weighed, and ground to fine powder using a cyclone mill (Cyclotec 1093, Foss, Höganäs, Sweden). Samples were analyzed for N, P, and K concentrations by near‐infrared reflectance spectroscopy. The concentrations were derived from previously established calibration models by recording a specific reflectance spectrum of each sample from 1250 to 2350 nm at intervals of 1 nm intervals (algorithmically averaged over 24 measurements). For details see Klaus et al., 2011; Kleinebecker, Klaus, & Hölzel, 2011; Kleinebecker, Weber, & Hölzel, 2011; Klaus et al., 2013. All values for vegetation composition (e.g., functional group coverage), species abundance, species richness, and aboveground biomass were summed up and averaged over the 6 years to provide robust measurements of long‐term effects.

### 2.4 Plant functional traits

Seven different vegetative, generative, and phenological plant traits reflecting functional responses to disturbance, competition, fecundity, and dispersal were chosen (see Table S1). As tall growth enables species to outcompete smaller ones for light (Weiher et al., 1999; Westoby et al., 2002) but requires constant investment in stem and plant tissue, *plant height* reflects the trade‐off between competition capacity (high relative growth rate) and immediate resistance to mowing and grazing disturbance due durable, tough tissue (Bernhardt‐Römermann et al., 2011) and may become too expensive if stress factors restrict photosynthesis (Givnish, 1995). *Specific leaf area* (SLA) and *leaf dry matter content* (LDMC) were chosen as leaf‐economics traits (Hodgson et al., 2011). Species with high SLA are associated with higher photosynthetic capacity per unit leaf mass, higher leaf N concentrations, a faster leaf turnover, and faster growth rate, allowing for a flexible response to variable light and soil resources (Westoby et al., 2002) and a higher tolerance to shade and defoliation, that is, due to mowing or grazing. These species are usually highly productive species with high nutrient acquisition rates in productive environments, quickly investing nutrients in high‐quality biomass and growth (Díaz et al., 2007). Species with high LDMC, on the other hand, show higher leaf durability and lower decomposition rate, thus higher nutrient conservation rates and decreased palatability often occur in unproductive, nutrient‐poor, and rather undisturbed, unmanaged environments. These species are thus associated with a nutrient retention strategy, by investing resources in long‐lived, through tissue rather than fast growth (Fortunel et al., 2009; Garnier et al., 2001; Louault et al., 2005). *Seed mass* and *seed number* were chosen as generative traits. Both are closely linked to dispersal, fecundity, and establishment/regeneration success (Laughlin & Wilson, 2014; Thompson, Parkinson, Band, & Spencer, 1997). Small seeds are associated with large seed production, which enhances dispersal success in productive or often disturbed environments (Grime, 1979; Laughlin & Wilson, 2014), whereas heavier seeds are positively associated with seedling establishment under competition and stressful conditions (Grime, 1979; Westoby, Leishman, & Lord, 1996). The phenological traits *flowering onset* and *flowering duration* are mechanisms to increase plant–pollinator interactions and are associated with mowing and grazing disturbance and competition avoidance (Weiher et al., 1999).

All traits related to vegetative growth were obtained from the TRY database (Kattge et al., 2011). Missing entries and life history traits concerning seed characteristics and flowering phenology were compiled from open source trait databases LEDA and BIOLFLOR (Kleyer et al., 2008; Klotz, Kühn, & Durka, 2002). For species recorded as aggregates (e.g., *Poa pratensis* agg.), as well as for subspecies (e.g., *Poa pratensis* subsp. *angustifolia*—unless the trait database had data for these subspecies), traits of the superordinate species (e.g., *Poa pratensis*) were compiled. Missing values in these databases were either selected from other literature or, if not obtainable otherwise, were extrapolated using the mean of the genus in the overall species pool of both databases, representing just 14% of a total of 361 species. In the case of six species, the missing data could not be gap‐filled and were thus omitted.

### 2.5 Statistical analysis

For statistical analyses, vegetation datasets were adapted by deleting tree and shrub entries in order to avoid great data distortions in life history traits of grassland species. We deleted a total of 14 species (mostly trees) with a mean maximum species coverage of 1.19% and an absolute maximum species coverage of 7% (*Juniperus communis*, in one year). Relative coverage in percent of the plant functional groups “graminoids,” “forbs,” and “legumes” was extracted from the vegetation records, summing up to 100%, and averaged over the six‐year period. We calculated the Shannon Diversity Index (HS; Spellerberg & Fedor, 2003). Functional traits were standardized, and community‐weighted mean trait values (CWM) were calculated for each year by averaging and weighing by their respective abundance over all species in a community (Garnier et al., 2007). Functional diversity (FD) was calculated for each trait separately via Rao′s quadratic entropy equation (Rao's *Q*), as Rao′s *Q* takes into account species abundance, dissimilarity, and evenness in trait space, without being correlated with species richness (Lepš, de Bello, Lavorel, & Berman, 2006; Mouchet, Villéger, Mason, & Mouillot, 2010; Rao, 1982). Functional composition (CWM) and functional diversity (via Rao's quadratic entropy) were computed using R (R Version 1.0.143; R Core Team 2016). Except soil characteristics, all data were averaged over the sampling period from 2008 to 2013.

General patterns of land‐use type and intensity, nutrient stoichiometry and plant traits in multidimensional space were explored by principal component analysis (PCA) using PCORD, Version 6.08 (McCune & Mefford, 2011). The PCA was performed with z‐transformed data.

In order to determine the different drivers of each plant trait, several linear mixed effect models (LMER) were calculated. As interest lay in analyzing supraregional patterns, and not testing regional effects, the study region was included in all models as a random factor. Using the LMER function implemented in the lme4 R package (Bates et al., 2015; R Version 1.0.143), CWM and Rao's *Q* of each trait were defined as a function of a set of categorical and continuous variables. As edaphic factors, soil pH and soils (being either organic, groundwater‐dependent peat soils, or mineral soils) were included. Management intensity (mowing, grazing, and fertilization), plant biomass nutrient stoichiometry (N:P, C:N), aboveground biomass, the Shannon Diversity index were also included.

For the calculation of the relative importance (explained variance) of single factors or factor groups on trait means (CWM) and diversity (via Rao's *Q*), residual sum of squares was obtained by performing ANOVA type III calculations. For the calculations of marginal R² values, the method presented by Nagakawa and Schielzeth (2013) was used.

In order to achieve normal distribution of data, SLA_Rao_ and flowering duration_Rao_ were root transformed. All other CWM and FD data except SLA_CWM_, flowering onset_CWM_ and flowering duration_CWM_ were log transformed. Linear mixed effect models with integrated backward step function were calculated in order to reduce the number of influencing factors and to identify the main driving ones. For model selection, the Akaike information criterion (AIC) was applied. Model assumptions were checked using the Shapiro–Wilk test for normality of residuals and diagnostic plots for controlling linearity and heteroskedasticity.

Single nutrient availability and its impact on functional traits were assessed via Spearman correlation analyses, whereas nutrient limitation and its impact on functional traits were examined using nutrient ratios in our LMER modeling. Furthermore, to check for intercorrelation among variables, a Spearman rank correlation for the full data set was calculated.

## 3 RESULTS

The studied grasslands showed great variation in their environmental and vegetation characteristics (for detailed information see Table 2). The gradient in land‐use intensity is reflected by fertilization level (ranging from 0–218.15 kg N × ha^−1^ × year^−1^), mowing frequency (0–4 times a year), and grazing intensity (0–947.9 LU × days × ha^−1^ × year^−1^). Ratios of Carbon: Nitrogen (C:N), Nitrogen:Phosphorus (N:P), and Nitrogen:Potassium (N:K) in aboveground biomass also varied strongly (13.3–36.43; 5.55–9.95; and 0.64–5.29, respectively). Average species richness ranged from 12.5 to 62.4 species on 16 m^2^, and the amount of biomass ranged from 27.9 to 441.5 g per m^2^.

**Table 2:** Mean values and respective standard errors of the analyzed variables and parameters, calculated for a time period of 6 years. Units: height = cm; specific leaf are = mm^2^/mg; leaf dry matter content = mg/mg; seed mass = mg; seed number = none; flowering onset = month; flowering duration = months

Variables	MV	*SE*	Min	Max	Parameters	MV	*SE*	Min	Max
**Functional composition (CWM)**					**Edaphic**				
Height	0.367	0.006	0.211	0.674	Soil depth	58.060	2.692	11.000	107.000
Specific leaf area	0.450	0.003	0.338	0.528	Soil pH	6.515	0.059	4.580	7.450
Leaf dry matter content	0.466	0.005	0.374	0.665					
Seed mass	0.031	0.001	0.012	0.080	**Land use and stoichiometry**				
Seed number	0.021	0.003	0.002	0.256	Land‐use intensity (LUI)	1.642	0.045	0.500	3.270
Flowering onset	0.762	0.002	0.693	0.849	Fertilization	1.000	0.110	0.000	6.580
Flowering duration	0.312	0.004	0.208	0.430	Mowing	1.000	0.069	0.000	3.020
					Grazing	1.000	0.106	0.000	8.890
**Functional Diversity (Rao's *Q*)**					C	43.518	0.033	42.567	44.584
Height	0.021	0.001	0.008	0.079	N	2.157	0.026	1.458	3.192
Specific leaf area	0.009	0.000	0.002	0.024	P	0.293	0.003	0.206	0.376
Leaf dry matter content	0.039	0.002	0.008	0.113	K	2.091	0.041	1.050	3.138
Seed mass	0.002	0.000	0.000	0.014	C:N ratio	20.599	0.244	13.744	30.239
Seed number	0.008	0.001	0.000	0.102	N:P ratio	7.375	0.064	5.937	9.543
Flowering onset	0.009	0.000	0.004	0.026	N:K ratio	1.109	0.032	0.616	2.491
Flowering duration	0.015	0.000	0.004	0.037					
					**Vegetation**				
					Species diversity	28.636	0.829	13.222	66.444
					Shannon diversity index	2.330	0.039	1.150	5.816
					Biomass	233.763	7.019	28.719	439.119
					Herb cover	29.048	0.819	4.147	56.824
					Legume cover	9.782	0.583	0.000	32.565
					Graminoid cover	60.801	1.091	22.962	93.331

### 3.1 General patterns in functional composition and functional diversity

**Figure 1:**
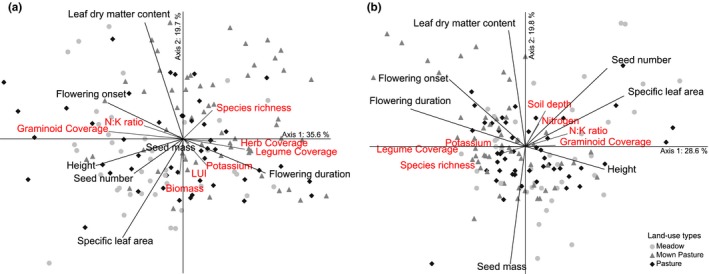
PCA ordination plot of (a) functional composition (CWM) and (b) functional diversity (FD). Red vectors point in the direction of increasing values for the respective edaphic land use, stoichiometric, or plant compositional variables with longer vectors indicating stronger correlations between vectors and axes. PCA axis eigenvalues for (a) (1) 2.49, (2) 1.38; Cut‐off *r* ^2^ = .180; and (b) (1) 2.00; (2) 1.38; Cut‐off *r* ^2^ = .180

Ordination analyses of trait CWM and Rao's *Q* revealed complex gradients of functional characteristics, species diversity, land‐use intensity, and plant stoichiometry. The first two PCA axes explained in sum the greatest amount of total variation, which were cumulative 55.3% and 48.4% of total variation in CWM and Rao's *Q*, respectively (Figure 1a,b).

For CWM, the first PCA axis reflected functional group composition and was positively correlated with legume and forb coverage and negatively with graminoid coverage (Figure 1a). Flowering onset, vegetation height, and seed number showed a negative loading with the first axis, whereas flowering duration showed a positive loading. The second axis was positively associated with LDMC and negatively with SLA. The first axis was orientated toward less intensively used pastures. In contrast, the second, being related to low SLA CWM, was orientated toward low land use, characterized by less intensive mowing and fertilization intensity (Table S2), associated with low plant biomass production and decreased potassium concentration.

For Rao's *Q*, the first PCA axis reflected functional group composition and represented plots with high mowing and low grazing values (Figure 1b). Legume coverage was positively associated with phenological trait functional Rao's *Q* (flowering duration and less with flowering onset); all showing a negative loading with the first axis. Graminoid coverage, however, showed a positive loading on the first axis, and was positively associated with leaf‐economy traits (SLA and height) and seed number. Along the first axis, plots were distributed according to land‐use type, with high‐intensely used meadows on the negative and high‐intensely used meadows on the positive end. The second PCA axis was positively correlated with land‐use intensity (via biomass N concentrations and N:K ratios) and soil depth (Table S2), being orientated toward intensively used meadows. Species diversity was negatively correlated with land‐use intensity. LDMC Rao's *Q* was positively correlated with the second axis, pointing toward rather low‐intensively used pastures, whereas seed mass Rao's *Q* showed a negative loading with the second axis.

### 3.2 Drivers of trait‐specific functional composition and functional diversity

#### 3.2.1 CWM and Rao's *Q* LMER modeling

**Figure 2:**
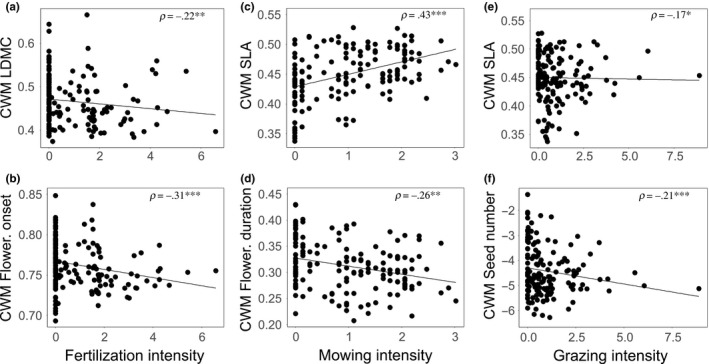
Pairwise correlations between trait community‐weighted means (CWM) and land‐use parameters. Fertilization intensity and (a) CWM leaf dry matter content (LDMC) and (b) flowering onset; mowing intensity and (c) CWM‐specific leaf area (SLA) and (d) flowering duration; grazing intensity and (e) CWM SLA and (f) seed number. Spearman correlation values (ρ) are given. Asterisks and letters indicate respective significance values: *p *>* *.5 = n.s.; .5 > *p* > .1 = *; .01 > *p* > .1 = **; .01 < *p* = ***

**Figure 3:**
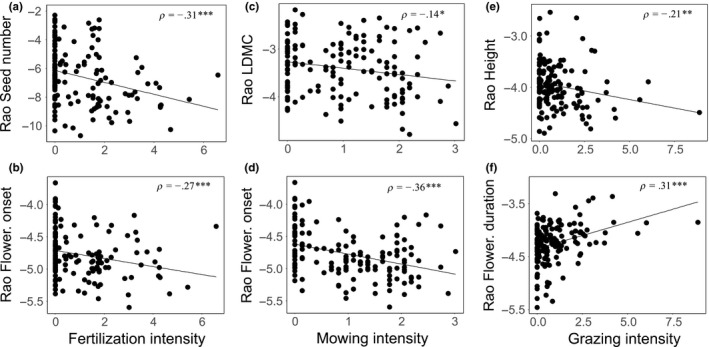
Pairwise correlations between trait functional diversity (Rao's Q) and land‐use parameters. Fertilization intensity and (a) Rao seed number and (b) Rao flowering onset; mowing intensity and (c) Rao leaf dry matter content (LDMC) and (d) Rao flowering onset; grazing intensity and (e) Rao height and (f) Rao flowering duration. Spearman correlation values (ρ) are given. Asterisks and letters indicate respective significance values: *p* > .5 = n.s.; .5 > *p* > .1 = *; .01 > *p* > .1 = **; .01 < *p* = ***

Linear mixed effect models explained between 6.4% and 49.5% of the variation in trait‐specific CWMs and Rao's *Q* values. Land‐use type affected all analyzed trait groups or syndromes. Fertilization promoted species with decreased seed production and seed mass variability (Seed mass Rao's *Q*). Grazing, on the other hand, promoted small‐growing species with decreased SLA and decreased seed number, but increased flowering duration. LDMC, flowering onset, and flowering duration variability was increased in grazed grasslands, whilst seed number variability was decreased (Table 3, Figures 2 and 3).

**Table 3:** Summary of linear mixed effect (LMER) models of (A) trait‐specific functional community‐weighted means (CWM) and (B) trait‐specific functional diversity (FD). Functional community‐weighted means and functional diversity of traits were modeled as function of land‐use, stoichiometric, vegetation, and edaphic parameters, respectively. Soils consist of two categories “mineral soils” and “organic peat soils”; region consisted of three categories: “Schwäbische Alb,” “Hainich‐Dün,” and “Schorfheide‐Chorin”. Significance levels are given below

	Height			SLA			LDMC			Seed number		
	*n*	Marg *R* ^2^	Cond *R* ^2^	*n*	Marg *R* ^2^	Cond *R* ^2^	*n*	Marg *R* ^2^	Cond *R* ^2^	*n*	Marg *R* ^2^	Cond *R* ^2^
	150	0.401	0.40	150	0.445	0.45	150	0.309	0.45	150	0.065	0.277
	Estim.	*t* Value	Sign.	Estim.	*t* Value	Sign.	Estim.	*t* Value	Sign.	Estim.	*t* Value	Sign.
(A)												
Intercept	↓	−8.444	***	↑	8.26	***		−10.92	***	↓	−14.88	***
Land use												
Fertilization Grazing	↓	−4.37	***	↓	−2.281	*				↓	−3.58	***
Stoichiometry												
CN				↓	−4.70	***	↑	3.37	***			
NP	↑	2.979	**	↓	−4.27	***	↑	5.83	***			
Vegetation												
Species diversity	↓	−3.346	**				↓	−2.06	*			
Biomass	↑	5.954	***	↑	5.87	***						
Edaphics												
Soil type							(org)↑	2.79	**			
Soil pH	↑	3.424	***									

	Height			SLA			LDMC			Seed number		
	*n*	Marg *R* ^2^	Cond *R* ^2^	*n*	Marg *R* ^2^	Cond *R* ^2^	*n*	Marg *R* ^2^	Cond *R* ^2^	*n*	Marg *R* ^2^	Cond *R* ^2^
	145	0.099	0.10	145	0.361	0.38	150	0.064	0.59	150	0.065	0.277
	Estim.	*t* Value	Sign.	Estim.	*t* Value	Sign.	Estim.	*t* Value	Sign.	Estim.	*t* Value	Sign.
(B)												
Intercept	↓	−10.925	***	↑	26.49	***	↓	−8.22	***		−1.44	
Land use												
Fertilization												
Grazing							↑	2.21	*	↓	−3.09	**
Stoichiometry												
CN	↑	0.03	2.30	*			↓	−3.53	***			
NP	↑	0.10915	2.409	*								
Vegetation												
Species diversity												
Biomass				↑	3.03	**				↓	−3.57	***
Edaphics												
Soil type				(org)↑	7.96	***						
Soil pH	↑	2.807	**							↓	−1.98	*

	Seed mass			Flower. onset			Flower. duration		
	*n*	Marg *R* ^2^	Cond *R* ^2^	n	Marg *R* ^2^	Cond *R* ^2^	*n*	Marg *R* ^2^	Cond *R* ^2^
	145	0.495	0.496	150	0.349	0.352	150	0.406	0.484
	Estim.	*t* Value	Sign.	Estim.	*t* Value	Sign.	Estim.	*t* Value	Sign.
(A)									
Intercept	↓	−13.97	***	↑	14.94	***	↑	27.09	***
Land use									
Fertilization Grazing							↑	3.49	***
Stoichiometry									
CN	↑	5.34	***	↑	4.88	***			
NP	↑	4.51	***	↑	2.80	**			
Vegetation									
Species diversity	↑	2.45	*						
Biomass	↑	2.57	*				↓	−3.49	***
Edaphics									
Soil type	(org)↓	−3.69	***	(org)↑	4.74	***	(org)↓	−7.92	***
Soil pH	↑	3.67	***	↑	4.88	***			

	Seed mass			Flower. onset			Flower. duration		
	*n*	Marg *R* ^2^	Cond *R* ^2^	*n*	Marg *R* ^2^	Cond *R* ^2^	*n*	Marg *R* ^2^	Cond *R* ^2^
	150	0.174	0.351	150	0.145	0.157	145	0.301	0.333
	Estim.	*t* Value	Sign.	Estim.	*t* Value	Sign.	Estim.	*t* Value	Sign.
(B)									
Intercept	↓	−14.09	***	↓	−25.81	***	↑	11.76	***
Land use									
Fertilization	↓	−2.07	*						
Grazing				↑	3.32	**	↑	3.60	***
Stoichiometry									
CN	↑	3.09	**						
NP									
Vegetation									
Species diversity	↑	2.80	**	↑	2.17	*			
Biomass	↑	3.80	***	↓	−2.41	*			
Edaphics									
Soil type							(org) ↓	−4.59	***
Soil pH							↓	−2.70	**

**Figure 4:**
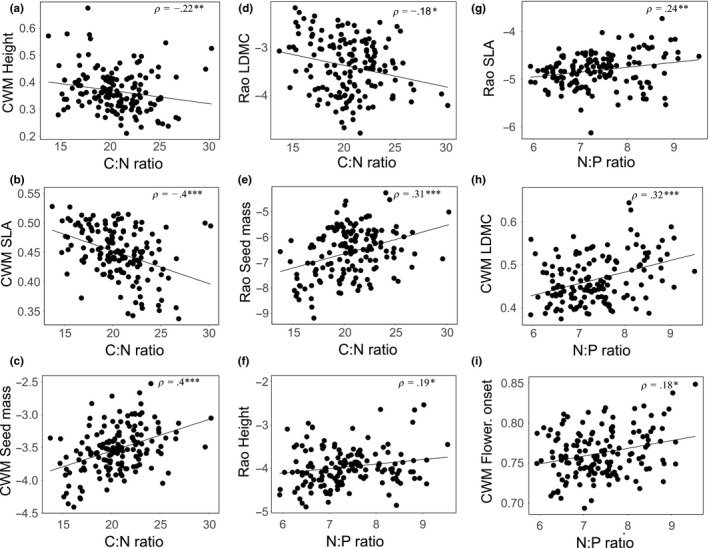
Pairwise correlations between functional composition (CWM) and functional diversity (Rao's Q) and nutrient stoichiometry. Carbon: Nitrogen ratio (C:N) and (a) CWM height and (b) CWM‐specific leaf area (SLA) and (c) CWM seed mass and (d) Rao leaf dry matter content (LDMC) and (e) Rao seed mass. Nitrogen:Phosphorus ratio (N:P) and (f) Rao height and (g) Rao SLA and (h) CWM LDMC. N:P ratio and (i) CWM flowering onset. Spearman correlation values (ρ) are given. Asterisks and letters indicate respective significance values: *p* > .5 = n.s.; .5 > *p* > .1 = *; .01 > *p* > .1 = **; .01 < *p* = ***

Nutrient stoichiometry affected all analyzed trait groups. Communities with large C:N and N:P ratios consisted of species with increased height variability, lower SLA, and larger LDMC values, producing bigger or heavier seed and flowering later in the year. Large N:P ratios additionally promoted taller growing species. Large C:N ratios, on the other hand, promoted species with reduced LDMC variability and increased variability in seed mass (Table 3; Figure 4).

Our analyses showed that species diversity affected leaf economics, generative, and phenological traits between species. In more diverse, that is, species‐rich systems, plant communities rather consisted of smaller growing species with reduced LDMC, but increased seed mass values. More productive communities, with higher aboveground biomass production, consisted of taller growing species with higher and more variable SLA and seed mass values, whilst mean flowering duration was decreased. Furthermore, these communities also consisted of species with more similar seed number and flowering onset (Table 3).

Our models indicate that edaphic factors significantly affected traits of all analyzed trait groups or syndromes. Soils with increased pH values harbored communities with taller and more variably growing species, producing heavier seed and with delayed flowering onset. Variability in seed number, however, as well as variability in flowering duration was decreased. Organic soils were positively associated with LDMC values, whilst mean seed mass and SLA variability were decreased. Plant communities on organic soils, furthermore, started flowering later in the year and over a shorter, less variable period of time (Table 3).

#### 3.2.2 Functional composition and functional diversity model variance partitioning

**Figure 5:**
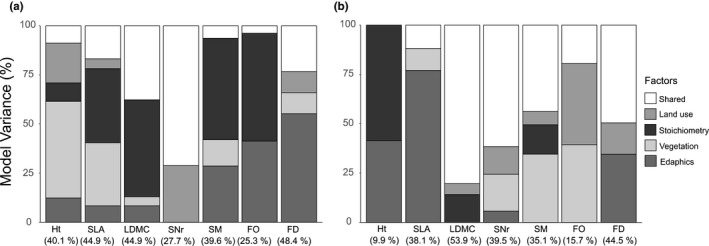
Shares of environmental factors on total explained variance of functional composition (CWM) and functional diversity (Rao's Q) modelling. Shares were upscaled to 100%; total variability explained by each model is given below trait names. Environmental factors stand for themselves or were summarized in factor groups: Edaphics = Soil depth, pH; Land use = fertilization, grazing; Stoichiometry = C:N ratio, N:P ratio; Vegetation = species number, Shannon Index, biomass; Shared = variability explained by more than one factor or factor group

Our models showed variable and trait‐specific effects of all environmental factors (Figure 5). However, the overall pattern revealed that foliar nutrient stoichiometry and land use explain a greater share of observed variance of trait CWMs than species richness and community productivity (i.e., biomass production). Interestingly, species richness and biomass production explain a greater share of observed variance of trait Rao's *Q* in generative traits and SLA, whereas land use and nutrient stoichiometry explain a greater share of observed variance in height, LDMC, and flowering duration. Whilst nutrient stoichiometry overall explained a larger proportion of variance in functional composition (CWM) and diversity (Rao's *Q*) of leaf economics and generative traits, land use better explained the observed variance in functional composition and diversity in generative and phenological traits (Figure 5).

## 4 DISCUSSION

Our study found functional composition (CWM) and functional diversity (FD via Rao's *Q*) of specific plant functional traits to be significantly related to land use, nutrient stoichiometry, species richness, production of aboveground plant biomass, and edaphic factors across 150 temperate grasslands. Generally, both nutrient stoichiometry and land use explained more variation in trait data than species richness and grassland productivity together. However, it was discernible that land use explained a greater share of observed variation in functional composition and functional diversity of competition‐related traits, whereas nutrient stoichiometry did so in addition to traits related to nutrient retention and stress avoidance. As emerging patterns are of high complexity, we discuss all sets of factors separately below.

### 4.1 Land‐use type and intensity

Our analyses indicate that land use affected all analyzed trait groups, corroborating our initial hypothesis. Hypothesis 1 was partly confirmed, as land use explained the greatest share of trait variation in trait functional diversity (Rao's *Q*) of competition‐related traits, but unexpectedly it also did so in trait functional composition (CWM). However, it did not explain the greatest share of observed variation in all analyzed traits as we expected.

The response of vegetation composition to land use depends on the interaction of its components, with some overriding, mitigating, or reinforcing the effects of others, depending on how the specific trait focused on is controlled by the suite of environmental drivers (Socher et al., 2012; Völler, Auge, Bossdorf, & Prati, 2013). In our study, frequently mown plots were highly fertilized (Spearman ρ = 0.648, *p* < .01), whereas intensively grazed plots were, if at all, only rarely mown (Spearman ρ = −0.713, *p* < .01). Hence, mowing was excluded from our LMER modeling, but due to the strong correlations, we discuss mowing intensity in line with fertilization intensity and contrasting grazing intensity.

Grazing, a temporarily and spatially variable form of disturbance, showed the strongest effects of land‐use types on all analyzed traits in our LMER models. Our results indicate that grazing promotes stress avoiding species with “slow, retentive strategies” that retain nutrients by investing in long‐lived, supportive structures with increased variability and reduced seed output. Mowing, however, affects plant species traits antagonistically, by stimulating the growth of highly competitive species with “fast, acquisitive strategies,” quickly absorbing and investing nutrients in (re)growth of palatable tissues after biomass removal (Louault et al., 2005; Pakeman et al., 2009).

Heavy, nonselective grazing pressure has been frequently associated with the promotion of small‐growing species with a rosette or stoloniferous architecture and a fast regrowth of high‐quality, very palatable tissue (Díaz et al., 2007; Westoby, 1998). This strategy is indicative of a grazing tolerance, as species are able to cope with frequent destruction of aboveground biomass. Under drier conditions or moderate, selective grazing, however, a grazing avoidance strategy is more useful. Prostrate and perennial, slow‐growing species, investing in the production of tough, long‐lived, and rather unpalatable tissues are promoted under these conditions (Díaz et al., 2007; Pakeman, 2004).

Especially in the absence of fertilization, grazing is also associated with net nutrient loss through continuous biomass removal. Under these conditions, the investment of reduced resources in less, but heavier seed may be advantageous as heavier seed may be more successful in germinating and establishing under competitive and stressful conditions (Grime, 1979; Westoby et al., 1996). Mowing can be associated with nutrient loss, if cut biomass is continuously removed from sites that are not fertilized and if the removal of biomass exceeds nutrient input from the atmosphere. In our study, however, intensely mown plots are also fertilized, hence providing plant communities with a good supply of nutrients and reducing competition by removing standing biomass. Species with acquisitive nutrient strategies are promoted—quickly absorbing available nutrients, investing them into high production of biomass by quickly resprouting and regrowing after biomass removal (Louault et al., 2005; Pakeman et al., 2009). The effects of fertilization (and along with it, partly also mowing) on functional composition (CWM) reflect a trade‐off between investment in tall and productive growth—advantageous under competitive environmental conditions—and the costs of maintaining green and supportive tissue under repetitive biomass removal (Bernhardt‐Römermann et al., 2011; Klimešová, Janeček, Bartušková, Lanta, & Doležal, 2010; Pausas & Lavorel, 2003).

In our study, intensely grazed plant communities, furthermore, consisted of species with extended and variable flowering phenological traits. Flowering seasonality has been shown to change under land use, mowing regimes leading to earlier flowering onset, whilst grazing promoting later flowering species (Reisch & Poschlod, 2009). This can be explained by community composition, as high‐intensity mowing promotes plant communities dominated by early flowering graminoid species, whereas low‐intensity mowing or grazing predominantly promotes forbs with later flowering onsets and longer flowering durations. Greater variability in flowering onset and duration, as well as extended flowering, are advantageous under disturbed conditions, as plant species reduce the loss of resources through removal of reproductive organs through grazing or mowing and also reduce pollinator competition (Reisch & Poschlod, 2009; Vamosi et al., 2006).

The consideration of land‐use effects on functional diversity on one hand, and on species diversity and productivity (biomass production) on the other hand, allows a more detailed view on community assembly patterns. Fertilization and mowing were associated with a decrease in species diversity and an increase in biomass production. This indicates that under increased fertilization, competitive species with “acquisitive strategies” that best exploit high N availability will enhance biomass production. The induced competition for light displaces less competitive species; fewer species with similar leaf‐economy trait expressions, and thus reduced trait differences between them, remain. This leads to community‐wide trait convergence (Grime, 2006; Mayfield & Levine, 2010). Regular mowing, on the other hand, reduces standing aboveground biomass and thus competition for light, providing more ecological niches whilst also tightening ecological filters. This allows less competitive species with different trait expressions to prevail and grow after biomass removal, permitting species niche differentiation and promoting trait diversity (Grime, 2006; Mayfield & Levine, 2010; Velbert, Kleinebecker, Mudrak, Schwartze, & Hölzel, 2017).

Land‐use intensity as a sum of fertilization, grazing, and mowing intensities was, as hypothesized, positively correlated with leaf‐economy traits, increasing the investment in a competition‐related strategy. We were also partly able to show a positive correlation between leaf‐economy and generative investment (see Lienin & Kleyer, 2011), mainly expressed by increased seed output by species with tall growth and high SLA values. The investment in generative traits at high land‐use intensity was decreased, whilst the expression of acquisitive strategies in leaf‐economy traits increased with increasing land‐use intensity, indicating a clear trade‐off between vegetative growth and reproduction. At first sight, this contrasts the results from Lienin and Kleyer (2011), who found greatest reproductive investment in highly disturbed and nutrient‐rich environments on one hand, favoring fast‐growing annuals with high‐seed output, and in rather undisturbed, nutrient‐poor environments on the other hand, favoring slow‐growing perennials producing many light or few heavy seeds. Lienin and Kleyer (2011), however, considered a much longer disturbance‐resource gradient compared to our study. The sites studied here were located rather in “the middle” of this axis and spanning a shorter gradient.

At intermediate disturbance and resource availability levels, Lienin and Kleyer (2011) also observed lowest regenerative investment, which is in line with our results.

### 4.2 Nutrient stoichiometry and edaphics

In line with our hypothesis, nutrient stoichiometry played an important role in many aspects of functional composition (CWM) of all analyzed trait syndromes; whilst the impact on Rao's *Q* was limited to plant height, LDMC, and seed mass. Nutrient stoichiometry explained a greater share of variation compared to land use in CWM data of traits related to nutrient retention and nutrient use, whereas land use had greater explanatory power in competition and competition–avoidance‐related traits. We observed variable impacts of nutrient stoichiometry on leaf‐economy, generative, and phenological traits depending on the relative limiting nutrient.

According to the leaf‐economics hypothesis proposed by Wright (Wright et al., 2004), nutrient‐poor conditions (N shortage, and even more so, P shortage portrayed by wide C:N and N:P ratios, respectively) are associated with low SLA, high LDMC, and low productivity (Fujita et al., 2014; Lienin & Kleyer, 2011; Pakeman et al., 2009). By predominantly producing supportive tissue with high contents of C‐rich lignin and other recalcitrant compounds, tough, durable, and slow‐growing leaves, these species display a retentive, conservative growth strategy with low biomass production (Suter & Edwards, 2013; Westoby & Wright, 2006).

Observed patterns of the promotion of tall‐growing species under nutrient‐poor conditions (portrayed by wide N:P ratios) seem to be contradictory at first sight. Especially in the study area Schorfheide‐Chorin, many of the assessed plots are located on drained fen soils. These plots are characterized by nutrient poor and slightly acidic soils, containing large amounts of organic matter and being strongly influenced by CO_3_‐rich ground water, which binds phosphorus, thus making it unavailable for plants (Afif, Matar, & Torrent, 1993). These unfertilized but mainly mown plots are characterized by tall growing, productive plant communities producing lighter seed, which is characteristic for a nutrient acquisitory, competitive strategy. LDMC‐rich tissues may be produced by tall growing, productive upper sward species such as *Arrhenatherum elatius, Glyceria fluitans*, or *Phalaris arundinace*a. Furthermore, the explanatory power of CWM height is by far the lowest of all models. Height generally shows great intraspecific variability, strongly dependent of local environmental conditions. It hence seems likely that in these mesic temperate grasslands, soil moisture may play a bigger role on plant community functionality than nutrient input. We therefore suggest putting great care into the interpretation of height to avoid overestimation of effects on this trait.

Our results, corroborated also by other studies (i.e., Fujita et al., 2014; Lienin & Kleyer, 2011), show that higher N:P ratios were associated with a conservative investment in reproduction by producing heavier seed. Under nutrient‐stress or highly competitive conditions (i.e., under N and P shortage), successful seedling recruitment and better seedling performance are essential for plant species to ensure their persistence. This can be attained by a trade‐off between growth and reproduction strategies and by partitioning available resources (Fujita et al., 2014; Güsewell, 2004). By exhausting available P and shifting investment from many light to few heavy seeds, stable P concentrations in diaspores are provided (Fujita et al., 2014; Tautenhahn et al., 2008), allowing for successful seedling establishment and persistence under competitive conditions.

Effects on phenological traits indicated a promotion of competition‐ and disturbance‐avoiding strategies. Under nutrient shortage, minimizing resource investment in reproductive organs, and maximizing successful pollination and seed production, is crucial for species to persist. The shift toward a delayed flowering onset may reflect an adaptation response to early management, especially mowing or grazing. By delaying flowering and seed production after the first cut of the year, nutrient losses due to biomass harvest are reduced (Reisch & Poschlod, 2009; Vamosi et al., 2006).

### 4.3 Vegetation diversity and productivity

We hypothesized that plant species richness and productivity are closely related to leaf‐economy traits, but also to generative and phenological traits. Our LMER results did corroborate this by showing that species richness and productivity both affect all analyzed trait syndromes. Species‐rich communities consist of species which increasingly invest in reproduction by increasing seed mass, presumably to enhance the chance of diaspore establishment (Grime, 1979). Greater variability in phenological traits in species‐rich communities indicates pollination competition avoidance and increases chances of successful pollination and seed set (Kwak, Velterop, & van Andel, 1998). Increased nutrient availability, which is directly associated with land‐use intensity (Blüthgen et al., 2012; Klaus et al., 2011) and graminoid dominance, promoted the investment in competition‐related leaf‐economy traits, increasing biomass production and leading to species loss. In line with our results, productivity has often been found to be negatively associated with plant species richness in real‐world agricultural grasslands (Koerselman & Meuleman, 1996; Socher et al., 2012; Verhoeven, Koerselman, & Meuleman, 1996).

Under nutrient‐poor conditions, due to heterogeneous resource availability and ecological niche differentiation, interspecific competition is reduced or “evened out” and allows for more species to coexist (Laliberté, Norton, Scott, & Mason, 2013; Niinemets & Kull, 2005; Tilman & Pacala, 1993). This leads to increased species numbers and decreased biomass production (Fujita et al., 2014; Klaus et al., 2011).

## 5 CONCLUSION

Our analyses indicate that land use, nutrient availability, species richness, and plant functionality interact via a complex network. Increased land‐use intensity clearly promoted species with acquisitive strategies through increased nutrient availability. Our results also point out that land use (type and intensity) better explained variation in functional diversity (Rao's *Q*), whilst nutrient stoichiometry was rather related to variation of functional composition (CWM). These results might suggest that nutrient stoichiometry plays a role in community assembly by coarsely sorting species in “acquisitive strategists” and “retentive strategists,” whereas stress and disturbance administrated by land use fine‐tune community assembly within these two strategy groups by sorting species into available ecological niches along the land‐use gradient. This work highlights the importance of considering both land use and nutrient availability to understand the mechanisms behind observed community assembly and vegetation response patterns to management and disturbance regimes, also showing the importance of considering both species diversity and functional diversity (FD) when looking at grassland management.

## CONFLICT OF INTEREST

None declared.

## AUTHOR'S CONTRIBUTIONS

VB, VK, TK conceived the idea for the manuscript; VB, TK, CP defined the final analyses; VB analyzed the data and outlined previous versions of the manuscript; VB, VK, DS, JM, SS, SB, DP, NH, MF contributed data; YN, JP gave valuable input on previous versions of the manuscript; and all authors contributed on the finalization of the manuscript.
