# Supplementary material for: Nutrient stoichiometry and land use rather than species richness determine plant functional diversity
Source: Ecol Evol. 2017 Dec 3;8(1):601–16. doi: 10.1002/ece3.3609 (PMC5756835; doi:10.1002/ece3.3609)
Supplement: Supplementary file 1 [file ECE3-8-601-s001.docx]

|  |  |  |  |  |  |  |  |
| --- | --- | --- | --- | --- | --- | --- | --- |
| Traits, trait attributes and abbreviations | |  | Unit | Ecological Relevance | Source | Literature | |
|  |  |  |  |  |  |  |  |
| Vegetative | Vegetative Height | Ht | m | RGR, competition ability, biomass/light caputre | LEDA, TRY | Weiher et al 1999, Lavorel & Garnier 2002 |  |
|  | Specific Leaf Area | SLA | mm²/mg | RGR, resource availability and disturbance | LEDA, TRY | Gross et al 2007, DeJong & Doyle 1985, |  |
|  |  |  |  | response, photosynthetic capacity, C-fixation |  | Lavorel & Garnier 2002 |  |
|  | Leaf Dry Matter Content | LDMC | mg/g | RGR, predictor of plant response to resource | LEDA, TRY | Gross et al 2007 |  |
|  |  |  |  | availability and disturbance |  |  |  |
|  |  |  |  |  |  |  |  |
| Generative | Seed Mass | SM | mg | Fecundity, regeneration, establishment, dispersal | LEDA, Biolflor | Westoby 1998 |  |
|  | Seed Number | SNr | - | Fecundity, regeneration, establishment, dispersal | LEDA |  |  |
|  |  |  |  |  |  |  |  |
|  | Flowering onset | FO | months | dispersal, disturbance/competition avoidance | LEDA | Pakeman 2004 |  |
|  | Flowering duration | FD | months | dispersal, disturbance/competition avoidance | LEDA |  |  |
|  |  |  |  |  |  |  |  |
|  |  |  |  |  |  |  |  |

**Table S1:** Selected vegetative and generative traits and their ecological relevance. All trait name abbreviations, their units are given. The respective online trait data base, as well as additional literature, is indicated.
