# Supplementary material for: Nutrient stoichiometry and land use rather than species richness determine plant functional diversity
Source: Ecol Evol. 2017 Dec 3;8(1):601–16. doi: 10.1002/ece3.3609 (PMC5756835; doi:10.1002/ece3.3609)
Supplement: Supplementary file 2 [file ECE3-8-601-s002.docx]

|  |  |  |  |  |  |  |  |  |  |  |  |  |  |  |  |  |  |  |  |
| --- | --- | --- | --- | --- | --- | --- | --- | --- | --- | --- | --- | --- | --- | --- | --- | --- | --- | --- | --- |
| **A) Principal Component Analysis Functional composition** |  |  |  |  |  |  |  |  |  |  | **B) Principal Coordinate Analysis Functional Diversity** |  |  |  |  |  |  |  |  |
|  |  |  |  |  |  |  |  |  |  |  |  |  |  |  |  |  |  |  |  |
| **CWM Eigenvectors** |  |  |  |  |  |  |  |  |  |  | **FD Eigenvectors** |  |  |  |  |  |  |  |  |
| Axis |  | a(1) |  |  | a(2) |  |  | a(3) |  |  |  | a(1) |  |  | a(2) |  |  | a(3) |  |
|  |  |  |  |  |  |  |  |  |  |  |  |  |  |  |  |  |  |  |  |
| Specific leaf area |  | -0.575 |  |  | -0.698 |  |  | -0.204 |  |  |  | 0.680 |  |  | 0.288 |  |  | -0.481 |  |
| Leaf dry matter content |  | -0.363 |  |  | 0.821 |  |  | -0.133 |  |  |  | -0.116 |  |  | 0.664 |  |  | 0.323 |  |
| Height |  | -0.828 |  |  | -0.188 |  |  | 0.252 |  |  |  | 0.546 |  |  | -0.130 |  |  | -0.479 |  |
| Seed number |  | -0.467 |  |  | -0.243 |  |  | -0.078 |  |  |  | 0.565 |  |  | 0.447 |  |  | -0.025 |  |
| Seed mass |  | 0.057 |  |  | -0.032 |  |  | 0.963 |  |  |  | -0.104 |  |  | -0.671 |  |  | -0.290 |  |
| Flowering onset |  | -0.715 |  |  | 0.254 |  |  | 0.348 |  |  |  | -0.524 |  |  | 0.383 |  |  | -0.650 |  |
| Flowering duration |  | 0.783 |  |  | -0.242 |  |  | 0.256 |  |  |  | -0.791 |  |  | 0.214 |  |  | -0.341 |  |
|  |  |  |  |  |  |  |  |  |  |  |  |  |  |  |  |  |  |  |  |
|  |  |  |  |  |  |  |  |  |  |  |  |  |  |  |  |  |  |  |  |
| **Environmental parameters** |  |  |  |  |  |  |  |  |  |  | **Environmental parameters** |  |  |  |  |  |  |  |  |
| Axis | a(1) | ρ | sign. | a(2) | ρ | sign. | a(3) | ρ | sign. |  | a(1) | ρ | sign. | a(2) | ρ | sign. | a(3) | ρ | sign. |
| Axis Eigenvectors | 2.593 |  |  | 1.593 |  |  | 1.093 |  |  |  | 2.593 |  |  | 1.593 |  |  | 1.093 |  |  |
|  |  |  |  |  |  |  |  |  |  |  |  |  |  |  |  |  |  |  |  |
| Soil depth | -0.294 | 0.000 | *** | -0.094 | 0.254 | n.s. | -0.325 | 0.000 | *** |  | 0.327 | 0.000 | *** | 0.449 | 0.000 | *** | 0.033 | 0.692 | n.s. |
| Soil pH | -0.247 | 0.002 | ** | 0.222 | 0.006 | ** | 0.236 | 0.004 | ** |  | 0.142 | 0.084 | n.s. | 0.116 | 0.156 | n.s. | 0.136 | 0.098 | n.s. |
| Fertilization | -0.073 | 0.377 | n.s. | -0.340 | 0.000 | *** | -0.153 | 0.061 | n.s. |  | 0.019 | 0.822 | n.s. | -0.101 | 0.220 | n.s. | 0.185 | 0.023 | * |
| Mowing | -0.340 | 0.000 | *** | -0.318 | 0.000 | *** | -0.110 | 0.181 | n.s. |  | 0.337 | 0.000 | *** | -0.093 | 0.260 | n.s. | 0.067 | 0.416 | n.s. |
| Grazing | 0.410 | 0.000 | *** | 0.009 | 0.918 | n.s. | -0.133 | 0.105 | n.s. |  | -0.374 | 0.000 | *** | 0.128 | 0.118 | n.s. | -0.031 | 0.710 | n.s. |
| Land-use intensity (LUI) | 0.019 | 0.814 | n.s. | -0.449 | 0.000 | *** | -0.306 | 0.000 | *** |  | -0.073 | 0.376 | n.s. | 0.024 | 0.773 | n.s. | 0.129 | 0.117 | n.s. |
| C | -0.180 | 0.028 | * | 0.299 | 0.000 | *** | 0.425 | 0.000 | *** |  | 0.187 | 0.022 | * | -0.203 | 0.013 | * | -0.172 | 0.035 | * |
| Ca | 0.302 | 0.000 | *** | -0.077 | 0.352 | n.s. | 0.033 | 0.693 | n.s. |  | -0.216 | 0.008 | ** | 0.052 | 0.527 | n.s. | -0.090 | 0.272 | n.s. |
| K | 0.355 | 0.000 | *** | -0.384 | 0.000 | *** | -0.154 | 0.060 | n.s. |  | -0.370 | 0.000 | *** | 0.043 | 0.603 | n.s. | 0.067 | 0.417 | n.s. |
| Mg | -0.159 | 0.053 | n.s. | -0.128 | 0.119 | n.s. | -0.232 | 0.004 | ** |  | 0.241 | 0.003 | ** | 0.241 | 0.003 | ** | -0.022 | 0.785 | n.s. |
| N | -0.264 | 0.001 | *** | -0.117 | 0.153 | n.s. | -0.403 | 0.000 | *** |  | 0.207 | 0.011 | * | 0.262 | 0.001 | *** | 0.045 | 0.582 | n.s. |
| P | -0.073 | 0.373 | n.s. | -0.258 | 0.001 | *** | -0.528 | 0.000 | *** |  | -0.001 | 0.986 | n.s. | 0.344 | 0.000 | *** | 0.167 | 0.041 | * |
| C:N | 0.257 | 0.001 | *** | 0.138 | 0.092 | n.s. | 0.418 | 0.000 | *** |  | -0.201 | 0.014 | * | -0.272 | 0.001 | *** | -0.052 | 0.529 | n.s. |
| N:P | -0.200 | 0.014 | * | 0.268 | 0.001 | *** | 0.068 | 0.407 | n.s. |  | 0.216 | 0.008 | ** | 0.049 | 0.548 | n.s. | -0.075 | 0.362 | n.s. |
| N:K | -0.350 | 0.000 | *** | 0.332 | 0.000 | *** | -0.035 | 0.672 | n.s. |  | 0.339 | 0.000 | *** | 0.068 | 0.407 | n.s. | -0.002 | 0.984 | n.s. |
| Species Number | 0.458 | 0.000 | *** | 0.309 | 0.000 | *** | 0.433 | 0.000 | *** |  | -0.371 | 0.000 | *** | -0.274 | 0.001 | *** | -0.112 | 0.174 | n.s. |
| Shannon Diversity | 0.444 | 0.000 | *** | 0.168 | 0.040 | * | 0.363 | 0.000 | *** |  | -0.331 | 0.000 | *** | -0.277 | 0.001 | *** | -0.214 | 0.009 | * |
| Biomass | -0.144 | 0.079 | n.s. | -0.467 | 0.000 | *** | -0.201 | 0.014 | ** |  | 0.128 | 0.118 | n.s. | -0.130 | 0.112 | n.s. | 0.029 | 0.721 | n.s. |
| Herb Coverage | 0.489 | 0.000 | *** | -0.163 | 0.047 | * | 0.159 | 0.052 | n.s. |  | -0.280 | 0.001 | *** | -0.094 | 0.252 | n.s. | -0.370 | 0.000 | *** |
| Graminoid Coverage | -0.720 | 0.000 | *** | 0.201 | 0.013 | * | -0.109 | 0.182 | n.s. |  | 0.451 | 0.000 | *** | 0.055 | 0.503 | n.s. | 0.238 | 0.003 | * |
| Legume Coverage | 0.611 | 0.000 | *** | -0.227 | 0.005 | ** | -0.020 | 0.812 | n.s. |  | -0.397 | 0.000 | *** | -0.006 | 0.940 | n.s. | -0.012 | 0.883 | n.s. |
|  |  |  |  |  |  |  |  |  |  |  |  |  |  |  |  |  |  |  |  |
|  |  |  |  |  |  |  |  |  |  |  |  |  |  |  |  |  |  |  |  |

**Table S2**: Summary of the trait, axis and environmental eigenvectors of the Principal Component analysis Ordination. A) CWM trait eigenvectors and B) FD trait eigenvectors scaled to their standard deviation, as well as the corresponding Spearman correlations between axis eigenvectors and environmental parameter eigenvectors, are given.
