# Supplementary material for: Nutrient stoichiometry and land use rather than species richness determine plant functional diversity
Source: Ecol Evol. 2017 Dec 3;8(1):601–16. doi: 10.1002/ece3.3609 (PMC5756835; doi:10.1002/ece3.3609)
Supplement: Supplementary file 3 [file ECE3-8-601-s003.docx]

# Spearman Correlations

CWM Specific Leaf Are_a

CWM Leaf Dry Matter Content CWM Heigh_t

CWM Seed number

CWMSeed ---- CWM Flowering onset CWM Flowering duratio_n _

| Variables | **n** | **p** | **p-value** | **sign.** | **p** | **p-value** | **sign.** | **p** | **p-value** | **sign.** | **p** | **p-value** | **sign.** | **p** | **p-value** | **sign.** | **p** | **p-value** | **sign.** | **p** | **p-value** | **sign.** |
| --- | --- | --- | --- | --- | --- | --- | --- | --- | --- | --- | --- | --- | --- | --- | --- | --- | --- | --- | --- | --- | --- | --- |
| **Land use** |  |  |  |  |  |  |  |  |  |  |  |  |  |  |  |  |  |  |  |  |  |  |
| **Fertilizatian** | 150 | 0.208 | 0.011 |  | 0.068 | 0.408 | **n.s.** | -0.030 | 0.714 | **n.s.** | -0.207 | 0.011 |  | -0.181 | 0.027 |  | -0.318 | 0.000 |  | -0.077 | 0.347 | **n.s.** |
| **Mowing** | 150 | 0.217 | 0.008 |  | 0.070 | 0.393 | **n.s.** | 0.211 | 0.010 |  | 0.090 | 0.272 | **n.s.** | -0.097 | 0.237 | **n.s.** | -0.059 | 0.472 | **n.s.** | -0.191 | 0.019 |  |
| **Grazing** | 150 | 0.121 | 0.141 | **n.s.** | -0.315 | 0.000 |  | -0.399 | 0.000 |  | -0.197 | 0.016 |  | -0.096 | 0.244 | **n.s.** | -0.286 | 0.000 |  | 0.317 | 0.000 |  |
| LUI | 150 | 0.381 | 0.000 |  | -0.136 | 0.098 | **n.s.** | -0.181 | 0.027 |  | -0.169 | 0.039 |  | -0.242 | 0.003 |  | -0.393 | 0.000 |  | 0.034 | 0.678 | **n.s.** |
| **Nutrient availability and stoichiometry** | | | | | | | | | | | | | | | | | | | | | | |
| C | 150 | -0.332 | 0.000 |  | 0.396 | 0.000 |  | 0.335 | 0.000 |  | 0.062 | 0.453 | **n.s.** | 0.292 | 0.000 |  | 0.394 | 0.000 |  | -0.230 | 0.005 |  |
| K | 150 | 0.448 | 0.000 |  | -0.353 | 0.000 |  | -0.285 | 0.000 |  | -0.172 | 0.035 |  | -0.073 | 0.375 | **n.s.** | -0.571 | 0.000 |  | 0.463 | 0.000 |  |
| N | 150 | 0.083 | 0.312 | **n.s.** | -0.121 | 0.142 | **n.s.** | 0.005 | 0.955 | **n.s.** | 0.019 | 0.818 | **n.s.** | -0.308 | 0.000 |  | -0.092 | 0.262 | **n.s.** | -0.235 | 0.004 |  |
| P | 150 | 0.355 | 0.000 |  | -0.290 | 0.000 |  | -0.279 | 0.001 |  | -0.055 | 0.507 | **n.s.** | -0.409 | 0.000 |  | -0.380 | 0.000 |  | 0.070 | 0.394 | **n.s.** |
| C:N | 150 | -0.091 | 0.270 | **n.s.** | 0.128 | 0.119 | **n.s.** | -0.012 | 0.882 | **n.s.** | -0.030 | 0.712 | **n.s.** | 0.313 | 0.000 |  | 0.095 | 0.250 | **n.s.** | 0.241 | 0.003 |  |
| N:P | 150 | -0.440 | 0.000 |  | 0.247 | 0.002 |  | 0.179 | 0.028 |  | -0.088 | 0.286 | **n.s.** | 0.081 | 0.322 | **n.s.** | 0.277 | 0.001 |  | -0.288 | 0.000 |  |
| N:K | 150 | -0.419 | 0.000 |  | 0.264 | 0.001 |  | 0.211 | 0.010 |  | 0.088 | 0.285 | **n.s.** | -0.076 | 0.352 | **n.s.** | 0.420 | 0.000 |  | -0.506 | 0.000 |  |
| P:K | 150 | -0.321 | 0.000 |  | 0.209 | 0.010 |  | 0.188 | 0.021 |  | 0.154 | 0.061 | **n.s.** | -0.145 | 0.077 | **n.s.** | 0.431 | 0.000 |  | -0.530 | 0.000 |  |
| **Vegetation composition** | | | | | | | | | | | | | | | | | | | | | | |
| **Species number** | **150** | -0.376 | 0.000 |  | -0.007 | 0.935 | **n.s.** | 0.005 | 0.948 | **n.s.** | -0.141 | 0.084 | **n.s.** | 0.371 | 0.000 |  | 0.012 | 0.884 | **n.s.** | 0.233 | 0.004 |  |
| **Biamass** | **150** | 0.449 | 0.000 |  | -0.058 | 0.483 | **n.s.** | 0.058 | 0.485 | **n.s.** | -0.009 | 0.911 | **n.s.** | -0.065 | 0.427 | **n.s.** | -0.231 | 0.004 |  | -0.127 | 0.121 | **n.s.** |
| **Herb Caverage** | **150** | -0.025 | 0.763 | **n.s.** | -0.495 | 0.000 |  | -0.143 | 0.080 | **n.s.** | -0.094 | 0.252 | **n.s.** | 0.124 | 0.131 | **n.s.** | -0.126 | 0.123 | **n.s.** | 0.166 | 0.042 |  |
| **Graminoid Caverage** | **150** | 0.005 | 0.950 | **n.s.** | 0.364 | 0.000 |  | 0.239 | 0.003 |  | 0.066 | 0.423 | **n.s.** | -0.042 | 0.612 | **n.s.** | 0.222 | 0.006 |  | -0.592 | 0.000 |  |
| **Legume Coverage** | **150** | 0.282 | 0.000 |  | -0.623 | 0.000 |  | -0.379 | 0.000 |  | -0.218 | 0.008 |  | -0.025 | 0.765 | **n.s.** | -0.478 | 0.000 |  | 0.523 | 0.000 |  |

Variables

# Raa Specific Leaf Area

**Raa Leaf Dry Matter C Rao Height**

**Rao Seed number**

**Raa Seed** ---- **Rao Flowering anset**

**Rao Flowering duration _**

|  | | **p** | **p-value** | **sign.** | **p** | **p-value** | **sign.** | **p** | **p-value** | **sign.** | **p** | **p-value** | **sign.** | **p** | **p-value** | **sign.** | **p** | **p-value** | **sign.** | **p** | **p-value** | **sign.** |
| --- | --- | --- | --- | --- | --- | --- | --- | --- | --- | --- | --- | --- | --- | --- | --- | --- | --- | --- | --- | --- | --- | --- |
| **Land use** |  |  |  |  |  |  |  |  |  |  |  |  |  |  |  |  |  |  |  |  |  |  |
| **Fertilizatian** | 150 | -0.382 | 0.000 |  | -0.167 | 0.042 |  | -0.149 | 0.069 | **n.s.** | -0.247 | 0.002 |  | -0.133 | 0.104 | **n.s.** | -0.047 | 0.569 | **n.s.** | 0.126 | 0.123 | **n.s.** |
| **Mowing** | 150 | -0.402 | 0.000 |  | -0.348 | 0.000 |  | -0.076 | 0.355 | **n.s.** | 0.033 | 0.687 | **n.s.** | -0.098 | 0.232 | **n.s.** | -0.193 | 0.018 |  | -0.116 | 0.158 | **n.s.** |
| **Grazing** | 150 | 0.110 | 0.181 | **n.s.** | 0.184 | 0.024 |  | -0.213 | 0.009 |  | -0.172 | 0.035 |  | -0.066 | 0.424 | **n.s.** | 0.245 | 0.003 |  | 0.303 | 0.000 |  |
| LUI | 150 | -0.397 | 0.000 |  | -0.195 | 0.017 |  | -0.320 | 0.000 |  | -0.213 | 0.009 |  | -0.207 | 0.011 |  | 0.012 | 0.882 | **n.s.** | 0.189 | 0.021 |  |
| **Nutrient availability and stoichiometry** | | | | | | | | | | | | | | | | | | | | | | |
| C | 150 | 0.128 | 0.119 | **n.s.** | 0.021 | 0.796 | **n.s.** | 0.322 | 0.000 |  | 0.069 | 0.405 | **n.s.** | 0.264 | 0.001 |  | -0.360 | 0.000 |  | -0.401 | 0.000 |  |
| K | 150 | -0.135 | 0.099 | **n.s.** | -0.053 | 0.521 | **n.s.** | -0.237 | 0.004 |  | -0.206 | 0.011 |  | -0.071 | 0.388 | **n.s.** | 0.347 | 0.000 |  | 0.547 | 0.000 |  |
| N | 150 | -0.145 | 0.076 | **n.s.** | 0.101 | 0.220 | **n.s.** | -0.152 | 0.063 | **n.s.** | -0.017 | 0.838 | **n.s.** | -0.200 | 0.014 |  | -0.029 | 0.724 | **n.s.** | -0.092 | 0.263 | **n.s.** |
| p | 150 | -0.212 | 0.009 |  | -0.090 | 0.271 | **n.s.** | -0.353 | 0.000 |  | -0.095 | 0.246 | **n.s.** | -0.341 | 0.000 |  | 0.213 | 0.009 |  | 0.251 | 0.002 |  |
| C:N | 150 | 0.153 | 0.061 | **n.s.** | -0.100 | 0.223 | **n.s.** | 0.154 | 0.059 | **n.s.** | 0.005 | 0.956 | **n.s.** | 0.197 | 0.016 |  | 0.014 | 0.862 | **n.s.** | 0.084 | 0.309 | **n.s.** |
| N:P | 150 | 0.150 | 0.067 | **n.s.** | 0.387 | 0.000 |  | 0.206 | 0.011 |  | -0.073 | 0.376 | **n.s.** | 0.134 | 0.103 | **n.s.** | -0.144 | 0.079 | **n.s.** | -0.310 | 0.000 |  |
| N:K | 150 | 0.082 | 0.318 | **n.s.** | 0.169 | 0.038 |  | 0.139 | 0.090 | **n.s.** | 0.103 | 0.209 | **n.s.** | -0.012 | 0.880 | **n.s.** | -0.281 | 0.000 |  | -0.477 | 0.000 |  |
| P:K | 150 | 0.041 | 0.620 | **n.s.** | 0.021 | 0.802 | **n.s.** | 0.088 | 0.284 | **n.s.** | 0.168 | 0.040 |  | -0.079 | 0.339 | **n.s.** | -0.310 | 0.000 |  | -0.477 | 0.000 |  |
| **Vegetation composition** | | | | | | | | | | | | | | | | | | | | | | |
| Species number | 150 | 0.452 | 0.000 |  | 0.446 | 0.000 |  | 0.246 | 0.002 |  | -0.092 | 0.262 | **n.s.** | 0.430 | 0.000 |  | 0.144 | 0.080 | **n.s.** | 0.229 | 0.005 |  |
| Biomass | 150 | -0.422 | 0.000 |  | -0.415 | 0.000 |  | -0.235 | 0.004 |  | -0.067 | 0.415 | **n.s.** | -0.021 | 0.796 | **n.s.** | -0.140 | 0.088 | **n.s.** | -0.039 | 0.633 | **n.s.** |
| **Herb Coverage** | **150** | 0.129 | 0.114 | **n.s.** | 0.333 | 0.000 |  | -0.050 | 0.542 | **n.s.** | -0.089 | 0.280 | **n.s.** | 0.270 | 0.001 |  | 0.281 | 0.000 |  | 0.126 | 0.125 | **n.s.** |
| **Graminoid CoveraQe** | **150** | -0.329 | 0.000 |  | -0.412 | 0.000 |  | -0.139 | 0.091 | **n.s.** | 0.022 | 0.792 | **n.s.** | 0.019 | 0.817 | **n.s.** | -0.489 | 0.000 |  | -0.480 | 0.000 |  |
| **Legume Coverage** | **150** | 0.184 | 0.024 |  | 0.120 | 0.142 | **n.s.** | -0.276 | 0.001 |  | -0.217 | 0.008 |  | 0.030 | 0.718 | **n.s.** | 0.242 | 0.003 |  | 0.557 | 0.000 |  |

**Table S3**: Spearman correlation matrix of all analyzed parameters with trait-specific functional community-weighted means (CWM) and functional diversity (Rao’s quadratic entropy). Correlations coefficients rho and p-value are given. Asterisks and letters indicate respective significance values: p > 0.5 = n.s.; 0.5 > p > 0.1 = *; 0.01 > p > 0.1 = **; 0.01 < p = ***. Significant correlations are marked in bold.
