# Supplementary material for: Nutrient stoichiometry and land use rather than species richness determine plant functional diversity
Source: Ecol Evol. 2017 Dec 3;8(1):601–16. doi: 10.1002/ece3.3609 (PMC5756835; doi:10.1002/ece3.3609)
Supplement: Supplementary file 4 [file ECE3-8-601-s004.docx]

Spearman Correlations

Fertilizatien Mewing Grazing LUI

| rho  **Edaphics** | | | **p-value** | **sign.** | rho | **p-value** | **sign.** | rho | **p-value** | **sign.** | rho | **p-value** | **sign.** |
| --- | --- | --- | --- | --- | --- | --- | --- | --- | --- | --- | --- | --- | --- |
| **Seils** 150 -0.124 | | | 0.132 | **n.s.** | 0.119 | 0.147 | **n.s.** | -0.170 | 0.038 |  | -0.069 | 0.398 | **n.s.** |
| **Seil reactien (pH)** 150 -0.025 | | | 0.766 | **n.s.** | 0.070 | 0.398 | **n.s.** | -0.187 | 0.022 |  | -0.130 | 0.113 | **n.s.** |
| Soil depth 150 -0.266 | | | 0.001 |  | -0.036 | 0.663 | **n.s.** | 0.091 | 0.266 | **n.s.** | -0.053 | 0.521 | **n.s.** |
| **Nutrients availability and stoichiometry** | | |  |  |  |  |  |  |  |  |  |  |  |
| **Carben** | 150 | -0.289 | 0.000 |  | -0.088 | 0.286 | **n.s.** | -0.237 | 0.003 |  | -0.434 | 0.000 |  |
| **Calcium** | 150 | 0.078 | 0.340 | **n.s.** | -0.021 | 0.801 | **n.s.** | 0.046 | 0.578 | **n.s.** | 0.058 | 0.480 | **n.s.** |
| **Petassium** | 150 | 0.282 | 0.000 |  | 0.020 | 0.805 | **n.s.** | 0.304 | 0.000 |  | 0.447 | 0.000 |  |
| **Magnesium** | 150 | 0.038 | 0.643 | **n.s.** | 0.147 | 0.073 | **n.s.** | -0.042 | 0.610 | **n.s.** | 0.114 | 0.166 | **n.s.** |
| **Nitrogen** | 150 | 0.287 | 0.000 |  | 0.277 | 0.001 |  | -0.016 | 0.843 | **n.s.** | 0.403 | 0.000 |  |
| **Phosphorus** | 150 | 0.326 | 0.000 |  | 0.170 | 0.038 |  | 0.210 | 0.010 |  | 0.525 | 0.000 |  |
| C:N | 150 | -0.295 | 0.000 |  | -0.277 | 0.001 |  | 0.002 | 0.984 | **n.s.** | -0.421 | 0.000 |  |
| N:P | 150 | -0.055 | 0.502 | **n.s.** | 0.062 | 0.454 | **n.s.** | -0.185 | 0.023 |  | -0.133 | 0.106 | **n.s.** |
| N:K | 150 | -0.104 | 0.206 | **n.s.** | 0.050 | 0.542 | **n.s.** | -0.226 | 0.005 |  | -0.210 | 0.010 |  |
| P:K | 150 | -0.093 | 0.260 | **n.s.** | 0.055 | 0.503 | **n.s.** | -0.218 | 0.007 |  | -0.194 | 0.017 |  |
| **Vegetation composition** |  |  |  |  |  |  |  |  |  |  |  |  |  |
| **Species number** | 150 | -0.261 | 0.001 |  | -0.371 | 0.000 |  | 0.077 | 0.348 | **n.s.** | -0.415 | 0.000 |  |
| **Species richness** | 150 | -0.187 | 0.022 |  | -0.287 | 0.000 |  | 0.068 | 0.410 | **n.s.** | -0.320 | 0.000 |  |
| **Biemass** | 150 | 0.385 | 0.000 |  | 0.308 | 0.000 |  | 0.042 | 0.607 | **n.s.** | 0.526 | 0.000 |  |
| **Herb Ceverage** | 150 | -0.132 | 0.107 | **n.s.** | -0.162 | 0.048 | **n.s.** | 0.135 | 0.099 | **n.s.** | -0.130 | 0.114 | **n.s.** |
| **Gramineid Ceverage** | 150 | 0.140 | 0.087 | **n.s.** | 0.252 | 0.002 |  | -0.283 | 0.000 |  | 0.090 | 0.271 | **n.s.** |
| **Legume Ceverage** | 150 | -0.094 | 0.255 | **n.s.** | -0.208 | 0.011 |  | 0.360 | 0.000 |  | 0.050 | 0.543 | **n.s.** |

**Table S4**: Spearman correlation matrix of all analyzed ecological parameters with land-use parameters fertilization, mowing, grazing, and land-use intensity index (LUI). Correlation coefficients rho and p-value are given. Asterisks and letters indicate respective significance values: p > 0.5 = n.s.; 0.5 > p > 0.1 = *; 0.01 > p > 0.1 = **; 0.01 < p = ***. Significant correlations are marked in bold.
