# Supplementary material for: Nutrient stoichiometry and land use rather than species richness determine plant functional diversity
Source: Ecol Evol. 2017 Dec 3;8(1):601–16. doi: 10.1002/ece3.3609 (PMC5756835; doi:10.1002/ece3.3609)
Supplement: Supplementary file 5 [file ECE3-8-601-s005.docx]

Spearman Correlations

Carbon Calcium Potassium Magnesium Nitrogen Phos[!horus

|  | | rho | **e-value** | **sign.** | rho | **e-value** | **sign.** | rho | **e-value** | **sign.** | rho | **e-value** | **sign.** | rho | **e-value** | **sign.** | rho | **e-value** | **sign.** |
| --- | --- | --- | --- | --- | --- | --- | --- | --- | --- | --- | --- | --- | --- | --- | --- | --- | --- | --- | --- |
| **Edaphics** |  |  |  |  |  |  |  |  |  |  |  |  |  |  |  |  |  |  |  |
| **Soils** | 150 | 0.109 | 0.184 | **n.s.** | -0.031 | 0.704 | **n.s.** | -0.415 | 0.000 |  | 0.487 | 0.000 |  | 0.522 | 0.000 |  | 0.276 | 0.001 |  |
| **Soil reaction (pH)** | 150 | 0.018 | 0.825 | **n.s.** | 0.208 | 0.011 |  | -0.146 | 0.074 | **n.s.** | 0.247 | 0.002 |  | 0.081 | 0.326 | **n.s.** | 0.046 | 0.575 | **n.s.** |
| **Seil depth** | 150 | -0.056 | 0.495 | **n.s.** | -0.240 | 0.003 |  | 0.017 | 0.841 | **n.s.** | 0.214 | 0.009 |  | 0.171 | 0.037 |  | 0.253 | 0.002 |  |
| **Vegetation composition** |  |  |  |  |  |  |  |  |  |  |  |  |  |  |  |  |  |  |  |
| **Species number** | 150 | 0.179 | 0.028 |  | 0.256 | 0.002 |  | -0.078 | 0.340 | **n.s.** | -0.274 | 0.001 |  | -0.493 | 0.000 |  | -0.544 | 0.000 |  |
| **Species richness** | 150 | 0.107 | 0.192 | **n.s.** | 0.369 | 0.000 |  | -0.091 | 0.268 | **n.s.** | -0.106 | 0.196 | **n.s.** | -0.327 | 0.000 |  | -0.435 | 0.000 |  |
| **Biomass** | 150 | -0.073 | 0.373 | **n.s.** | -0.385 | 0.000 |  | 0.411 | 0.000 |  | -0.143 | 0.081 | **n.s.** | 0.180 | 0.028 |  | 0.260 | 0.001 |  |
| **Herb CoveraQe** | 150 | -0.007 | 0.932 | **n.s.** | 0.592 | 0.000 |  | 0.007 | 0.929 | **n.s.** | 0.285 | 0.000 |  | -0.003 | 0.975 | **n.s.** | -0.104 | 0.205 | **n.s.** |
| **Graminoid Coverage** | 150 | 0.145 | 0.077 | n.s. | -0.519 | 0.000 |  | -0.239 | 0.003 |  | -0.076 | 0.354 | **n.s.** | 0.195 | 0.017 |  | 0.122 | 0.137 | **n.s.** |
| **Legume Coverage** | 150 | -0.307 | 0.000 |  | 0.122 | 0.137 | **n.s.** | 0.489 | 0.000 |  | -0.262 | 0.001 |  | -0.351 | 0.000 |  | -0.036 | 0.659 | **n.s.** |

**Table S5**: Spearman correlation matrix of all analyzed ecological parameters with plant tissue nutrient content, used as proxy for soil nutrient availability. Correlation coefficients rho and p-value are given. Asterisks and letters indicate respective significance values: p > 0.5 = n.s.; 0.5 > p > 0.1 = *; 0.01 > p > 0.1 = **; 0.01 < p = ***. Significant correlations are marked in bold.
